# Telemedicine in Germany During the COVID-19 Pandemic: Multi-Professional National Survey

**DOI:** 10.2196/19745

**Published:** 2020-08-05

**Authors:** Arne Peine, Pia Paffenholz, Lukas Martin, Sandra Dohmen, Gernot Marx, Sven H Loosen

**Affiliations:** 1 Department of Intensive Care and Intermediate Care University Hospital Rheinisch Westfaelische Technische Hochschule Aachen Aachen Germany; 2 Department of Urology, Uro-Oncology, Robot Assisted and Reconstructive Urologic Surgery University Hospital Cologne Cologne Germany; 3 Department of Medicine III University Hospital Aachen Aachen Germany; 4 Clinic for Gastroenterology, Hepatology and Infectious Diseases University Hospital Düsseldorf Medical Faculty of Heinrich Heine University Düsseldorf Düsseldorf Germany

**Keywords:** telemedicine, coronavirus, COVID-19, telehealth, SARS-CoV-2, pandemic, survey, medical professional, availability, acceptance, burden

## Abstract

**Background:**

In an effort to contain the effects of the coronavirus disease (COVID-19) pandemic, health care systems worldwide implemented telemedical solutions to overcome staffing, technical, and infrastructural limitations. In Germany, a multitude of telemedical systems are already being used, while new approaches are rapidly being developed in response to the crisis. However, the extent of the current implementation within different health care settings, the user’s acceptance and perception, as well as the hindering technical and regulatory obstacles remain unclear.

**Objective:**

The aim of this paper is to assess the current status quo of the availability and routine use of telemedical solutions, user acceptance, and the subjectively perceived burdens on telemedical approaches. Furthermore, we seek to assess the perception of public information quality among professional groups and their preferred communication channels.

**Methods:**

A national online survey was conducted on 14 consecutive days in March and April 2020, and distributed to doctors, nurses, and other medical professionals in the German language.

**Results:**

A total of 2827 medical professionals participated in the study. Doctors accounted for 65.6% (n=1855) of the professionals, 29.5% (n=833) were nursing staff, and 4.9% (n=139) were identified as others such as therapeutic staff. A majority of participants rated the significance of telemedicine within the crisis as high (1065/2730, 39%) or neutral (n=720, 26.4%); however, there were significant differences between doctors and nurses (*P*=.01) as well as between the stationary sector compared to the ambulatory sector (*P*<.001). Telemedicine was already in routine use for 19.6% (532/2711) of German health care providers and in partial use for 40.2% (n=1090). Participants working in private practices (239/594, 40.2%) or private clinics (23/59, 39.0%) experienced less regulatory or technical obstacles compared to university hospitals (586/1190, 49.2%). A majority of doctors rated the public information quality on COVID-19 as good (942/1855, 50.8%) or very good (213/1855, 11.5%); nurses rated the quality of public information significantly lower (*P*<.001). Participant’s age negatively correlated with the perception of telemedicine’s significance (ρ=–0.23; *P*<.001).

**Conclusions:**

Telemedicine has a broad acceptance among German medical professionals. However, to establish telemedical structures within routine care, technical and regulatory burdens must be overcome.

## Introduction

### Background

The global pandemic caused by the severe acute respiratory syndrome coronavirus 2 is creating a historic challenge for health care providers, patients, and societies throughout the world. Hospitals are drastically increasing intensive care capacities in an effort to contain the effects of the pandemic. However, staffing, technical, and infrastructural limitations are impeding progress in this regard. With an estimated intensive care unit (ICU) hospitalization rate of 5%, the pandemic quickly surpassed the global hospital care capacities [1]. In response to the crisis, new approaches are urgently needed to avoid a medical crisis.

**Figure 1:**
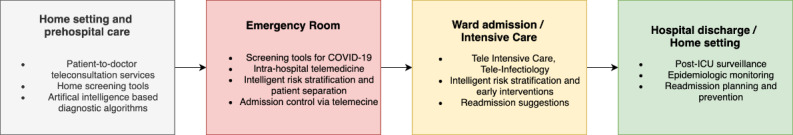
Levels of telemedical interventions during the COVID-19 crisis. COVID-19: coronavirus disease; ICU: intensive care unit.

To bring specialist care to the patients, diverse telemedical approaches have been implemented into patient care routine worldwide in both the ambulatory and hospital sectors (from the home setting to admission, treatment, and discharge), and adaptations have been developed for each use case (Figure 1).

### Telemedicine in Acute Emergency and Intensive Care

In the area of acute care medicine, specifically intensive care medicine, telemedicine has proven to be a success story. For example, the introduction of a remote intensivist program in two US tertiary care hospitals has led to a significant reduction of mortality (9.4% vs 12.9%; relative risk 0.73; 95% CI 0.55-0.95) and has proven to be cost-effective [2].

As a result of the worldwide shortage of medical professionals, not all patients are treated under the supervision of a specialized doctor. Although, it is estimated that, if specialized ICU physician staffing was implemented in nonrural US hospitals, approximately 53,000 lives and US $5.4 billion would be saved annually; as of 2010, only 10%-15% of the US ICUs were able to provide intensivist care, clearly a resource urgently needed in response to the coronavirus disease (COVID-19) [3]. Multiple studies have shown that providing a dedicated intensivist at an ICU leads to a significant reduction in mortality and reduces the length of stay [4,5]. Worldwide, telemedical tools have been rapidly adopted to the intensive care setting, providing specialist telemedical guidance to remote hospitals.

The advantages in the reduction of distance barriers between patients and physicians are also used to improve access to high-level intensive care in otherwise medically underserved areas. In adult intensive care wards, the introduction of telemedical surveillance by a specialized intensivist reduced severity-adjusted mortality by 33% and 30%, and the incidence of ICU complications by 44% and 50% in two intervention periods in an observational time series cohort study [6]. A recently published large retrospective study in the United States showed similar results as well for adult step-down or progressive care units, where patients in the telemedical intervention group had a survival benefit of 20% and had a significantly lower length of stay [7]. Consequently, telemedical solutions are also used for “in-house screening” of patients with COVID-19 (eg, by distributing tablet computers in emergency departments), minimizing the time of direct patient contact and, thus, cutting down the infection risk [8,9]. These findings were supported by a systematic review and meta-analysis of 13 studies involving 35 ICUs using a pre-post-design. The authors concluded that there was a reduction in ICU mortality (pooled odds ratio 0.80, 95% CI 0.66-0.97; *P*=.02) and a reduction in length of stay but stated that in-hospital mortality was not proven to be significantly reduced (pooled odds ratio 0.82, 95% CI 0.65-1.03; *P*=.08) [10].

Numerous worldwide experiences have shown that the formation of “telemedical excellence centers” is an efficient and fast way to provide telemedical specialist care to large populations. This is especially true for the reaction to global crises such as the coronavirus pandemic, as these telemedical centers can be created rapidly, concentrate specialist care locally, and deliver the highest quality care within large regions without travel restrictions or risk of infection for medical staff [11]. For example, in reaction to the pandemic and under support of the federal government of North-Rhine Westphalia in Germany, the University Hospitals of Aachen and Münster have built up a “virtual hospital” structure within weeks that provides a day-and-night availability of specialist intensivist and infectologist care for over 200 regional hospitals [12-14]. In China, the National Telemedicine Center of China in Zhengzhou has established a telemedicine-enabled outbreak alert and response network, connecting over 120 smaller hospitals [15]. In rapid response to the COVID-19 pandemic, a telemedical network was created in the Sichuan Province in Western China [11]. The first results of the retrospective success analysis showed telemedicine to be a “feasible, acceptable, and effective” way to provide health care and “allowed for significant improvements in health care outcomes.” Furthermore, entirely new, data-driven disease containment strategies using contact tracing–based mobile sensors were rapidly developed and established to track and impede chains of infection [16].

### Telemedicine in the Ambulatory Sector

In addition, within the ambulatory sector (eg, in home care and outpatient clinics), telemedical solutions are on the rise worldwide in response to the coronavirus crisis [17]. Over 50 US hospitals established or reinforced their telemedical health systems to allow clinicians to see patients who are at home without the risk of infection for medical professionals [8]. This might also be true for non–infection-related consultations (eg, in orthopedics [18] or chronic conditions [19]), reducing the need for repeated physical patient-physician contact and, consequently, the risk of cross-infections within a practice visit. A tool for the initial medical assessment of COVID-19 has been made available to German citizens by the German Central Institute for Statutory Health Insurance Physicians. The “COVID-Guide” is intended to enable patients to make an initial assessment of their own health situation in the event of possible complaints and uncertainties in connection with the coronavirus, which serves to accompany patients at home with telemedical means and, thus, recognize the occurrence of specific alarm symptoms at an early stage [20]. In contrast to other countries (eg, the Netherlands, the United Kingdom), German patients have generally free access to all medical specialties. A referral is not mandatory from the patient’s point of view. A patient can consult a specialist immediately and does not have to take a detour via a general practitioner. In the case of so-called family doctor–centered care, members of the statutory health insurance are bound to contract doctors of the health insurance companies when choosing a (telemedical) doctor. Telemedical consultations are mostly performed through direct patient-doctor contact.

To establish telemedicine in routine care, user acceptance by medical professionals is of the utmost importance to make effective use of telemedical resources. Furthermore, infrastructural problems (eg, broadband internet connectivity, organizational structures) are still thought to be a relevant factor hindering effective implementation of telemedical care [21]. To our knowledge, no structured, large-scale assessment of the perception of telemedical services within the crisis has been carried out accounting for differences between levels of care and medical professional groups. In this study, we examine the current status quo of the availability and routine use of telemedical solutions, the user acceptance, and the subjectively perceived burdens on telemedical approaches. Telemedical providers often serve as an additional source for medical knowledge. This is particularly important in crises, where the distribution of reliable information is key. Consequently, we assess the perception of the quality of public information among professional groups and their preferred information channels to evaluate efficient communication with the aforementioned providers.

## Methods

### Data Collection and Recruitment

Survey data collection took place on all days of the evaluated time frame between March 27, 2020, and April 11, 2020. Participants were acquired via numerous communication channels, taking into account the heterogeneous access and technical skills of different medical professional groups. Access (weblink) to the survey system was distributed through official communication channels and mailing lists of numerous medical societies and social media groups. Furthermore, several large- and medium-sized hospitals shared the weblink to the survey within their internal communication systems (eg, intranet platforms). The telemedical survey was part of a larger survey on coronavirus conducted within the time frame.

Data acquisition took place via a publicly accessible, web-based survey system (LimeSurvey, version 3.22.10; LimeSurvey GmbH) based on the programming language Hypertext Preprocessor (version 7.1.33, The PHP Group) and JavaScript (version 262, June 2017, ECMA International). The survey was accessible through all common web browsers as well as mobile phones.

All computational infrastructure was hosted on an Apache server (The Apache Software Foundation), while the system was physically hosted in Nuremberg, Germany to comply with all European data protection laws. No technical failure or server downtime was observed during the acquisition period. Data was stored using a My Structured Query Language (MySQL) database (Oracle Corporation).

### Statistical Analysis

All statistical preprocessing and analysis was carried out using the R statistical programming language [22] (Version 3.6.3, R Foundation for Statistical Computing) and the statistical software Jamovi (Version 1.2.16.0, The Jamovi Project) [23].

Appropriate sample size was calculated as described in Multimedia Appendix 1. A confidence level of 99% and an acceptable error margin of 3% were assumed [24].

The descriptive statistical data is laid out in total numbers as well as in relative percentages.

Assumption of normal distribution was tested using the Shapiro-Wilk test.

We used a nonparametic one-way analysis of variance to address potential differences between the professional groups. Post-hoc analysis was performed using Dwass-Steel-Chritchlow-Figner pairwise comparisons, when appropriate. Correlation analysis for ordinal variables was carried out using Spearman rho.

## Results

### Study Demographics and Survey Size

A total of 2927 participants took part in the survey within the observed time frame between March 27, 2020, and April 11, 2020. We calculated the ideal sample size for the survey to reach significance as described in the methods section. We assumed there were a total of 5.6 million medical professionals employed within the German health care domain, as reported by the German Federal Office of Statistics [25]. On a confidence level of 99% and within an acceptable error margin of 3%, the required sample rate was determined to be 1849 participants.

The median observed age was 43.0 (SD 11.8) years. Of the 2827 participants, 51.1% (n=1446) of participants identified as females, 47.6% (n=1345) identified as males, and 0.3% (n=69) chose not to disclose gender. The majority of the survey participants worked within a university hospital setting (n=1238, 43.8%), while 26.9% (n=749) worked within smaller regional hospitals. The ambulatory sector was represented by 21.6% (n=611) participants within private practices, 2.1% (n=60) within private clinics, and 1.6% (n=45) in a rehabilitation clinic setting. A total of 111 (3.9%) participants disclosed their work environment as “other” (eg, ambulatory nursing services). When asked about their profession, 65.6% (n=1855) identified as doctors, 29.5% (n=833) as nursing staff, and 4.9% (n=139) as other medical professionals such as therapeutic staff; Table 1).

**Figure 2:**
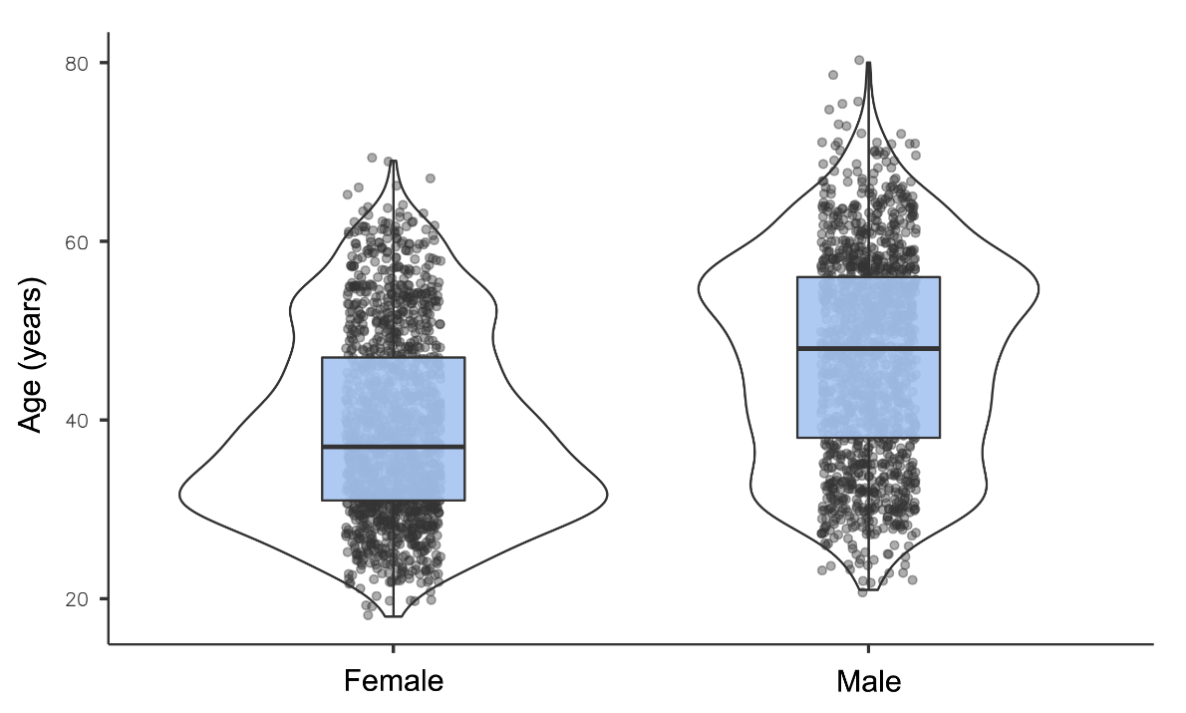
Age distribution split by gender within the study participants.

Visualization of the gender distribution already showed indications of a non-Gaussian distribution. We further assessed the pattern by performing a Shapiro-Wilk test, resulting in a statistic value of 0.98 (*P*<.001), thus confirming the hypothesis of a nonnormal distribution (Figure 2).

**Table 1:** Demographic data of the study population.

Variable		Value
Participant total, N		2827
Age (years), mean (SD)		43.0 (11.8)
**Gender, n (%)**		
	Female	1446 (51.1)
	Male	1345 (47.6)
	Missing/nondisclosed	69 (0.3)
**Work setting, n (%)**		
	University hospital, high level care	1238 (43.8)
	Regional hospital	749 (26.5)
	Ambulatory care/medical practice	611 (21.6)
	Private clinic	60 (2.1)
	Rehabilitation clinic	45 (1.6)
	Others (eg, ambulatory nursing service)	111 (3.9)
	Missing/nondisclosed	13 (0.4)
**Hospital environment, if working within hospital, n (%)**		
	Outpatient clinic	283 (10.0)
	Standard care ward	750 (26.5)
	Intensive care ward	486 (17.2)
	Operating theater	401 (14.2)
	Diagnostics	148 (5.2)
	Missing/nondisclosed	759 (27.8)
**Professional group, n (%)**		
	Doctors	1855 (65.6)
	Nursing staff	833 (29.5)
	Others (eg, therapeutic staff)	139 (4.9)

### Perception on the Use of Telemedicine and Telehealth Infrastructure

Participants were asked to estimate the significance of telemedicine during the coronavirus crisis (“How high do you estimate the significance of telemedicine and teleconsultations in the current crisis?”). Of the 2730 participants that responded to this question, the importance of telemedicine and teleconsultation was rated as high (n=1065, 39%) to neutral (n=720, 26.4%). The majority of the doctors (1036/1806, 57.4%), nurses (508/797, 63.8%), and other medical professionals (90/127, 70.9%) estimated the significance of telemedicine and teleconsultation within the COVID-19 crisis as either high (4) or very high (5).

A subcohort analysis of the different groups, however, showed significant (χ²_5_=15.8; *P*<.001) differences in the perception of telemedicine. In a subcohort analysis, differences between doctors and nurses (*W*=4.05, *P*=.01) as well as between doctors and others (*W*=4.36, *P*<.001) were shown to be significant, while differences between nurses and others did not reach statistical significance (*W*=2.62, *P*=.15).

**Figure 3:**
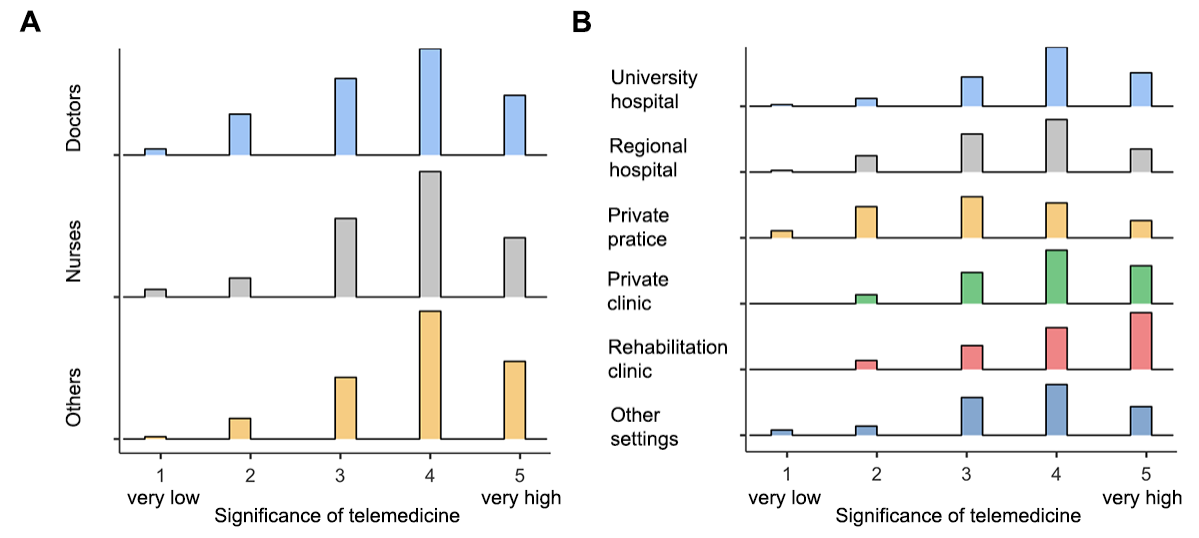
Subgroup analysis: relative significance of telemedicine among professional groups (A) and different work settings (B).

Closer analysis of the perception of telemedicine between health care professionals (Figure 3a) working in a diverse work environment (Figure 3b) revealed different response patterns within the observed groups. One difference was the significantly higher perception of telemedicine amongst health care workers within the stationary and hospital sector (χ²_5_=190; *P*<.001) compared to the ambulatory and practice sector, and a significantly higher estimation in university hospital workers (*W*=–18.39, *P*<.001) and regional hospital workers (*W*=–9.99, *P*<.001) compared to private practices (Table 2).

Further, correlation analysis revealed a significant negative correlation of the participant’s age with the perception of telemedicine’s significance (p=–0.23; *P*<.001).

**Table 2:** Significance of telemedicine in the coronavirus crisis.

Profession	How high do you estimate the significance of telemedicine in the current crisis?					
	1 (very low), n (%)	2, n (%)	3, n (%)	4, n (%)	5 (very high), n (%)	Total, n (%)
Doctors	38 (2.1)	255 (14.1)	477 (26.4)	664 (36.8)	372 (20.6)	1806 (100.0)
Nurses	21 (2.6)	52 (6.5)	216 (27.1)	345 (43.3)	163 (20.5)	797 (100.0)
Others	1 (0.8)	9 (7.1)	27 (21.3)	56 (44.1)	34 (26.8)	127 (100.0)
Total	60 (2.2)	316 (11.6)	720 (26.4)	1065 (39.0)	569 (20.8)	2730 (100.0)

### Availability of Telemedical Infrastructure and Establishment in Daily Routine

We further assessed the current availability of telemedical services during the pandemic. Despite being acknowledged as a significant tool during the COVID-19 crisis by the majority of participants, telemedicine was not yet part of the daily routine in most of the work environments. In only 20.2% (240/1189) of the university hospitals, 20.3% (12/59) of the private clinics, and 5.6% (41/726) of the regional hospitals telemedical consulting was in routine use. In contrast, 36% (214/595) of the participants working in a private practice setting already used telemedical tools routinely. Further analysis revealed a large proportion of hospitals that did not have access to telemedicine at all (405/1189, 34.1% for university hospitals and 393/726, 54.1% for regional hospitals; Table 3).

Furthermore, significance analysis and post hoc analysis revealed significant differences (χ²_4_=295, *P*<.001) between university hospitals and regional hospitals (*W*=–15.26, *P*<.001), between private practices and private clinics (*W*=–5.04, *P*=.01), and between private clinics and rehabilitation clinics (*W*=–5.88, *P*<.001).

To further investigate the reasons for the low use of telemedical tools, we addressed potential regulatory or technical obstacles within the participants’ work environment. Although participants working in private practices (239/594, 40.2%) or private clinics (23/59, 39.0%) experienced no regulatory or technical obstacles, most of the medical professionals working in university hospitals (586/1190, 49.2%) experienced at least partial obstacles. In total, only 22.7% (616/2711) answered the question “Do you experience regulatory or technical obstacles for telemedicine within you work environment?” with “yes” (Table 4).

**Table 3:** Availability of telemedicine in different medical settings.

Answer	Do you have the possibility to reduce direct patient contact by using telemedicine?						
	University hospital, n (%)	Regional hospital, n (%)	Private practice, n (%)	Private clinic, n (%)	Rehab clinic, n (%)	Others, n (%)	Total, n (%)
None at all	405 (34.1)	393 (54.1)	88 (14.8)	21 (35.6)	34 (77.3)	47 (48.0)	988 (36.4)
In rare cases	471 (39.6)	279 (38.4)	284 (47.7)	25 (42.4)	8 (18.2)	23 (23.5)	1090 (40.2)
Daily routine	240 (20.2)	41 (5.6)	214 (36.0)	12 (20.3)	1 (2.3)	24 (24.5)	532 (19.6)
Others	73 (6.1)	13 (1.8)	9 (1.5)	1 (1.7)	1 (2.3)	4 (4.1)	101 (3.7)
Total	1189 (100.0)	726 (100.0)	595 (100.0)	59 (100.0)	44 (100.0)	98 (100.0)	2711 (100.0)

**Table 4:** Subcohort analysis: regulatory or technical obstacles for telemedicine within different work environments.

Answer	Do you experience regulatory or technical obstacles for telemedicine within you work environment?						
	University hospital, n (%)	Regional hospital, n (%)	Private practice, n (%)	Private clinic, n (%)	Rehab clinic, n (%)	Others, n (%)	Total, n (%)
None	357 (30.0)	262 (36.1)	239 (40.2)	23 (39.0)	19 (43.2)	34 (34.7)	934 (34.5)
Partially	586 (49.2)	281 (38.7)	214 (36.0)	23 (39.0)	13 (29.5)	44 (44.9)	1161 (42.8)
Yes	247 (20.8)	183 (25.2)	141 (23.7)	13 (22.0)	12 (27.3)	20 (20.4)	616 (22.7)
Total	1190 (100.0)	726 (100.0)	594 (100.0)	59 (100.0)	44 (100.0)	98 (100.0)	2711 (100.0)

### Perception of Information Quality and Quantity

We assessed the perception of information quality and quantity within the different professional groups and the preferred communication channels to identify the most appropriate information strategy in a pandemic situation. Information was rated on a 5-point Likert scale, ranging from very poor (1) to very good (5). A majority of the participants rated the information quality either neutral (606/2827, 21.4%) or good (n=1341, 47.4%). Information quantity of public information concerning the coronavirus crisis was rated similar with neutral (981/2827, 34.7%) or good (n=1101, 38.9%). The detailed subgroup analysis, however, revealed that, although the majority of doctors rated the information quality as good (942/1855, 50.8%) or very good (n=213, 11.5%), a majority of the participating nurses rated the quality of public information lower on a Likert scale with 3 (neutral; 233/833, 28.0%) or 4 (good; n=330, 39.6%). When addressing the satisfaction on information quantity, the differences were smaller; 42.0% (779/1855) of the doctors and 33.4% (278/833) of nursing staff rated the information quantity with a 4 (“good”; Tables 5 and 6).

We performed a subcohort analysis to address the significance of differences between the professional groups. Differences between groups were significant for both quality (χ²_2_=69.3; *P*<.001) and quantity (χ²_2_=47.9; *P*<.001). Post hoc analysis with Dwass-Steel-Chritchlow-Figner pairwise comparisons showed significant differences in perception of information quality between doctors and nursing staff (*P*<.001), and nursing staff and other groups (*P*<.001), while differences between nurses and others were not shown to be significant. Addressing the information quantity, significant differences between doctors and nursing staff (*P*<.001), and doctors and others (*P*=.003) was shown, while the differences between nursing staff and other medical professionals were not significant (*P*=.96; Table 7).

We further assessed the main information sources for information within the COVID-19 pandemic. Out of the 2733 participants that responded to this question, a total of 83.6% (n=2284) of medical professionals used websites, 61.1% (n=1671) used television, and 30.7% (n=840) used newspapers within their top 3 sources of information on the COVID-19 pandemic. Social media was relatively low in use (n=388, 14.2%), and 34.6% (n=945) informed themselves through colleagues (Table 8).

**Table 5:** Perception of the information quality on the coronavirus among professional groups.

Professional group	How satisfied are you with the public information quality on coronavirus?					
	1 (very poor), n (%)	2, n (%)	3, n (%)	4, n (%)	5 (very good), n (%)	Total, n (%)
Doctors	119 (6.4)	235 (12.7)	346 (18.7)	942 (50.8)	213 (11.5)	1855 (100.0)
Nurses	61 (7.3)	165 (19.8)	233 (28.0)	330 (39.6)	44 (5.3)	833 (100.0)
Others	6 (4.3)	21 (15.1)	27 (19.4)	69 (49.6)	16 (11.5)	139 (100.0)
Total	186 (6.6)	421 (14.9)	606 (21.4)	1341 (47.4)	273 (9.7)	2827 (100.0)

**Table 6:** Perception of the information quantity on the coronavirus among professional groups.

Professional group	How satisfied are you with the public information quantity on coronavirus?					
	1 (very poor)	2	3	4	5 (very good)	Total
Doctors	19 (1.0)	195 (10.5)	697 (37.6)	779 (42.0)	165 (8.9)	1855 (100.0)
Nurses	30 (3.6)	219 (26.3)	225 (27.0)	278 (33.4)	81 (9.7)	833 (100.0)
Others	4 (2.9)	23 (16.5)	59 (42.4)	44 (31.7)	9 (6.5)	139 (100.0)
Total	53 (1.9)	437 (15.5)	981 (34.7)	1101 (38.9)	255 (9.0)	2827 (100.0)

**Table 7:** Post hoc Dwass-Steel-Critchlow-Fligner pairwise comparison.

Pairwise comparisons	Quality of coronavirus information			Quantity of coronavirus information	
	W	*P* value	W		*P* value
Doctors x nurses	–11.653	<.001	–9.252		<.001
Doctors x others	–0.139	>.99	–4.587		.003
Nurses x others	5.137	<.001	0.388		0.96

**Table 8:** Information sources about the pandemic used by participants; responses to the question "What are the top 3 sources you mainly use to inform yourself about the current coronavirus disease pandemic?" (N=2827).

Answer	n (%)
Websites	2284 (83.6)
Television	1671 (61.1)
Newspapers	840 (30.7)
Social media	388 (14.2)
Podcasts	563 (20.6)
Colleagues	945 (34.6)
Email newsletters	892 (32.6)
Mobile phone apps	191 (7.0)
Friends and relatives	265 (9.7)
Other sources	241 (8.8)

## Discussion

In reaction to the COVID-19 pandemic, telemedicine has emerged as a key technology to bring high-level medical care to patients while reducing the transmission of COVID-19 among patients, families, and clinicians. We conducted this study to assess the extent of the current implementation within different health care settings, user acceptance and perception, public information politics, and regulatory burdens that are potentially impeding implementation and consequently withholding lifesaving telemedical treatment from patients who are infected that require medical attention.

Within this study, we asked a total of 2927 German medical professionals about their perceptions of telemedicine and telehealth during the current coronavirus crisis. With Germany maintaining one of the largest health care sectors worldwide, this survey can also be seen as a blueprint for other industrialized nations with similar infrastructural resources.

The study received broad interests and was supported by numerous medical societies, quickly surpassing the required 1849 participants needed to reach the calculated study sample size. The perception of telemedicine during the current COVID-19 crisis was generally high throughout all professional groups. This is in line with several studies addressing acceptance of telemedical solutions, generally supporting the high significance in clinical routine for both nurses [26] and primary health care providers [27].

Almost all medical specialties and professional societies have developed COVID-19 specific telemedical approaches catered to their specific medical needs (eg, allergologist [28], neurologists [29], or urologists [30].

Another striking finding of our study, however, was the significantly higher perception of telemedicine among health care workers within the stationary or hospital sector compared to the ambulatory or practice sector during the current crisis. This might be the result of many telemedical apps designed specifically for the hospital setting (eg, tele-intensive care) and less apps for the home care or ambulatory setting. As a major result of our study, this should lead to further development of telemedical solutions for the ambulatory sector to contain infections, reduce unnecessary practice visits, and consequently decrease infection risk.

Interestingly, over one-third of private practices already had the possibility to reduce patient contact through telemedicine. In many cases, practitioners used makeshift and highly accessible tools, like messenger services to communicate with patients in a home setting [31].

However, in particular to the hospital setting, regulatory and technical burdens seem to be hindering the progress. This is particularly true for the public sectors, where a majority of the study participants reported at least occasional obstacles for telemedicine within their work environments. Reducing these obstacles as well as investing in technical infrastructure to provide optimal care for the patients harmed by COVID-19 within the crisis should be a priority for regulatory bodies and governments. It is, however, important to underline that only approximately one-fifth of the survey participants described a severe regulatory or technical hindering to telemedicine (answering “yes”).

Public information in particular plays a crucial role in the implementation of adherence to public health guidelines and fact-based communication toward peers and patients. Although a majority of health care providers rated the communication quality and quantity positively, a subcohort analysis highlighted a significantly lower perception for nurses and other medical personnel. As medical nurses and other medical professionals play an essential role in patient care during the crisis, specially targeted information material is urgently needed to address this deficiency. For example, special COVID-19–related information platforms are already in place (eg, within the American Nurses Association [32]). To reach the recipient, it is also important to note that most medical professionals used websites and television to keep informed, while social media only served a minor role for medical professionals. Interestingly, the general acceptance of telemedicine was negatively correlated with age. This could clearly be an indication of a targeted information gap concerning older medical professionals. It is important to underline that regulatory and technical aspects are not the only burdens hindering the establishment of telemedicine in routine care. In particular financial aspects, such as a lack of additional investment in infrastructure, expansion of the clinical staff, and additional training for clinical and administrative staff, can potentially impact implementation. Furthermore, social aspects, such as language barriers in direct audio-video conversation and lack of technical skills, can impede the progress in this regard. Regulatory aspects, such as the need for supplementary documentation, should be addressed early on to avoid the creation of an additional workload. The aforementioned aspects have to be taken into account when creating new telemedical infrastructures.

Clearly, this study has some limitations. First, the study population was limited to German-speaking participants. Larger studies with international participants have to be conducted to confirm the results. Second, due to the emergent nature of this survey, the professional groups were not evenly distributed within the participants, leading to potential implications for certain groups. This can potentially be overcome by a larger study, in which other medical professional groups are specifically targeted. Third, arising from the nature of online surveys, there was potentially more attraction for technophile participants, resulting in a distorted picture concerning things such as technical hurdles.

The COVID-19 pandemic is creating a historic global challenge for health care providers, patients, and societies alike. When technological, regulatory, and infrastructural burdens can quickly be overcome, telemedicine has the chance to transform from model implementations to a global supply structure, potentially saving thousands of patients’ lives.

## Appendix

Multimedia Appendix 1 Calculation of ideal sample size.

## Abbreviations

COVID-19: coronavirus disease
ICU: intensive care unit
MySQL: My Structured Query Language
