# Supplementary material for: Telemedicine in Germany During the COVID-19 Pandemic: Multi-Professional National Survey
Source: J Med Internet Res. 2020 Aug 5;22(8):e19745. doi: 10.2196/19745 (PMC7409912; doi:10.2196/19745)

Multimedia Appendix

**“Telemedicine in Germany during the COVID-19 Pandemic:**

**Multiprofessional National Survey”**

Figure 1: Calculation of ideal sample size, where z is the z score, ε is the margin of error, N is population size, p̂ is the population proportion.


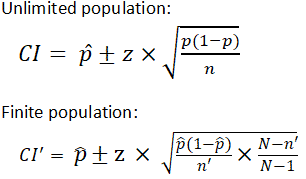

Supplement: Multimedia Appendix 1 [file jmir_v22i8e19745_app1.docx]
